# Intra-pyloric botulinum toxin injection improves liquid gastric emptying using ^99m^Tc DTPA scintigraphy: a case report in a 2 years- old girl with idiopathic gastroparesis

**DOI:** 10.22038/aojnmb.2025.84182.1599

**Published:** 2025

**Authors:** Khadijah R Sumitro, Andy Darma, Tri P Sucianti, Stepanus Massora, Alpha F Athiyyah, Reza G Ranuh, Subijanto M Sudarmo

**Affiliations:** 1 Department of Child Health, Faculty of Medicine, Universitas Airlangga, Surabaya, Indonesia; 2 Department of Child Health, Dr. Soetomo General Academic Hospital, Surabaya, Indonesia; 3 Division of Nuclear Medicine, Department of Radiology, Dr. Soetomo General Academic Hospital, Surabaya, Indonesia

**Keywords:** Pediatric, Gastroparesis, Gastric emptying scintigraphy Intra-pyloric botulinum toxin

## Abstract

Gastroparesis, characterized by delayed gastric emptying in the absence of mechanical obstruction, is a challenging condition to diagnose and treat in children due to limited pediatric-specific data. This case report presents a 15-month-old girl with recurrent and chronic vomiting since infancy, which worsened upon the introduction of solid foods. Initial diagnostic evaluations, including esophagogastroduodenoscopy (EGD) and upper gastrointestinal contrast study, ruled out structural abnormalities. A gastric emptying scintigraphy (GES) with ^99m^Tc DTPA confirmed significant gastric retention, leading to a diagnosis of idiopathic gastroparesis. Endoscopic intra-pyloric botulinum toxin injection (IPBI) was performed and resulting in significant symptom improvement. Post-procedure assessments revealed improved gastric emptying, with reduced retention at 60 and 180 minutes and a markedly decreased half-time (t_1/2_) was shown following the procedure. These findings highlight that IPBI may be a promising therapeutic option for pediatric idiopathic gastroparesis unresponsive to standard treatments. Further research is warranted to refine treatment protocols and evaluate long-term outcomes.

## Introduction

 Gastroparesis is characterized by delayed transit of gastric contents into the duodenum in the absence of mechanical obstruction. The diagnosis of gastroparesis is based on the presence of appropriate gastroparesis-associated symptoms or signs (including nausea, vomiting, early satiety, anorexia, weight loss, and epigastric pain) and the absence of obstruction structural lesion or ulceration in the gastric or small intestine, accompanied by delayed gastric emptying (1–3). Diagnosing gastroparesis in children is particularly challenging due to the lack of standardized diagnostic criteria, especially for younger patients, which makes the prevalence of pediatric gastroparesis unknown. Developing standardized clinical guidelines or treatment algorithms for gastroparesis in children is further complicated by limited therapeutic options and the need for individualized interventions based on patient-specific profiles (3, 4).

 Endoscopic intra-pyloric botulinum toxin injection (IPBI) has shown some benefit in treating adults with gastroparesis, but pediatric data remain limited (4). This report highlights the potential role of intra-pyloric botulinum toxin injection as a therapeutic option for pediatric patients with idiopathic gastroparesis who do not respond to the standard treatments. Additionally, the use of ^99m^Tc DTPA (Technetium-99m diethylene-triamine-pentaacetate) gastric emptying scintigraphy is emphasized as a valuable tool for both diagnosis and monitoring therapeutic response.

## Case report

 A 15-month-old girl was referred to a tertiary hospital with a history of recurrent vomiting since she was 3 months old. Her symptoms worsened at 6 months when she was introduced to solid food. Vomiting occurred more than 10 times per day and was reported consistently, not always associated with eating or drinking. Physical examination findings were unremarkable. She had a normal nutritional status with body weight of 10.6 kg and body height of 76 cm. According to the WHO 2006 anthropometric growth chart, her scores were 1< WAZ (weight for age z-score) <0, 0< HAZ (height for age z-score) <-1, and 1< WHZ (weight for height/ length z-score) <0 with normal nutritonal status. Empirical treatment with a proton pump inhibitor (PPI) and prokinetics (domperidone and erythromycin) was initiated, but her symptoms did not improve.

 An upper gastrointestinal (GI) contrast study revealed no anatomical obstruction from the esophagus to the pylorus. 

**Figure 1:**
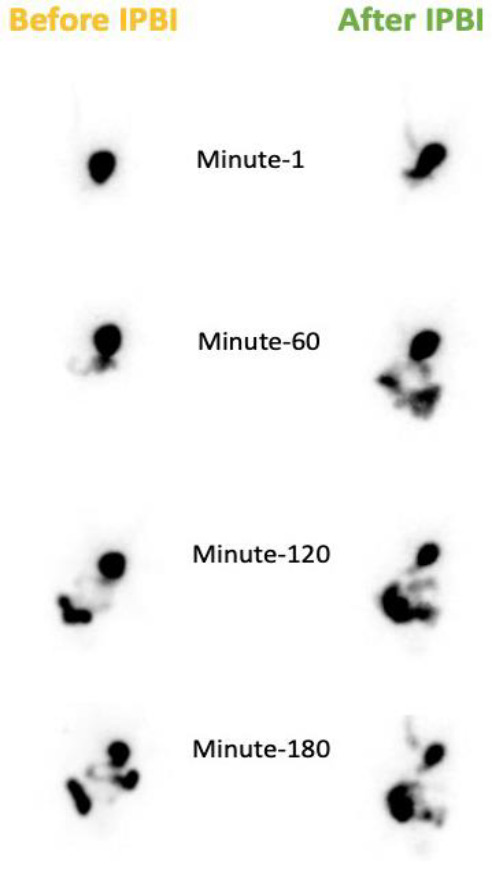
Gastric retention in 1, 60, 120 and 180 minutes from dynamic gastric emptying scintigraphy in left anterior oblique (LAO) position using ^99m^Tc DTPA (Technetium-99m diethylene-triamine-pentaacetate) (left: before IPBI; right: after IPBI)

 Esophagogastroduodenoscopy (EGD) showed pangastritis, and she was started on omeprazole and a mucosal protector. Thyroid function tests were within normal limits, with FT4 1.34 ng/dL (normal range 0.8–1.8 ng/dL) and TSH 3.34 mLU/L (normal range 0.8–8.2 mLU/L). Her symptoms persisted for nine months later and at 24 months, a gastric emptying scintigraphy (GES) was performed. A liquid gastric emptying study using ^99m^Tc DTPA (Technetium-99m diethylene-triamine-pentaacetate) showed a delayed gastric emptying (GE) with 93% and 36% retention in 60 and 180 minutes, respectively, and a half-time (t1/2) of 434 minutes (Figure 1). Her symptoms remained unresponsive to prokinetics and dietary modifications using nasogastric tube (NGT).

**Figure 2:**
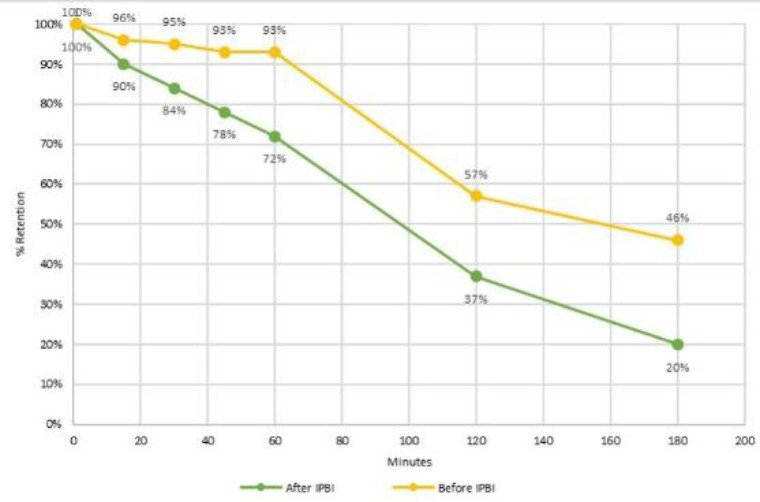
Improvement in gastric emptying (% gastric retention in minutes) after IPBI (**green line**) compared to before IPBI (**yellow line**)

 One month later, botulinum toxin (BTX) was injected endoscopically into the pylorus using a sclerotherapy needle at a dose of 6 units/kg up to a maximum of 100 units. The injection was distributed across four quadrants around the pylorus (25 U per quadrant). A vial containing 100 units of botulinum toxin was diluted in 1 milliliter of normal saline to create a 10-unit per 0.1 milliliter solution. Vomiting was dramatically improved and a follow-up scintigraphy evaluation two months later showed an improvement in GE, with 72% and 20% retention in 60 and 180 minutes, respectively, and t1/2 improved to 107 minutes (Figure 1). The improvement in gastric emptying (% gastric retention in minutes) before and after IPBI is detailed in Figure 2 and Table 1.

**Table 1:** Gastric Emptying Scintigraphy before and after intra-pyloric botulinum toxin injection (IPBI)

1^st^ Gastric Emptying Scintigraphy (Before IPBI)			2^nd^ Gastric Emptying Scintigraphy (After IPBI)		
Time	% Empty	% Retention	Time	% Empty	% Retention
15 mins	4%	96%	15 mins	10%	90%
30 min	5%	95%	30 min	16%	84%
45 mins	7%	93%	45 mins	22%	78%
60 mins	7%	93%	60 mins	28%	72%
120 mins	43%	57%	120 mins	63%	37%
180 mins	64%	46%	180 mins	80%	20%

## Discussion

 This case reported a girl with a history of recurrent vomiting. Gastroparesis commonly presents in children with symptoms of vomiting, abdominal pain, and nausea (5, 6). Diagnosing gastroparesis in children is challenging due to the scarcity of pediatric normative data and the lack of standardized diagnostic techniques. Several diagnostic methods have been explored, but many are time-consuming, costly, and infrequently used in routine clinical practice (3, 7). In this case, an upper GI contrast study and upper GI endoscopy or esophagogastroduodenoscopy (EGD) were performed to investigate the etiology of vomiting, both yielding normal results. The diagnosis of gastroparesis was based on the liquid gastric emptying study using ^99m^Tc DTPA (Technetium-99m diethylene-triamine-pentaacetate). The initial step in diagnosing gastroparesis is to exclude mechanical obstruction, which can be identified by upper gastrointestinal contrast imaging and EGD (8). An upper GI contrast study can identify any gastric or small bowel anatomical causes of obstruction. An upper GI endoscopy is performed to evaluate for gastric outlet obstruction and mucosal disease (3, 8, 9). Once mechanical obstructions are ruled out, the next step is to confirm the diagnosis of gastroparesis. Gastric emptying scintigraphy is considered the gold standard in diagnosing gastroparesis (8-11).

 Dietary intervention using a nasogastric tube and pharmacotherapy with domperidone and erythromycin were attempted to manage the vomiting. However, no significant symptomatic improvement was observed. Currently, no standardized therapy strategies or management algorithms exist for pediatric gastroparesis. The primary goals of gastroparesis treatment are symptom relief, correction of malnutrition, and restoration of sufficient oral intake of liquids and solid food (12). Nutrient liquids are often better tolerated than solids, as they empty from the stomach more quickly, within approximately 2–3 hours, and are less likely to be grossly impaired (12, 13). Data on dietary interventions in pediatric gastroparesis are limited and yield variable results. Improved gastric emptying was documented in an older, small trial involving preterm and low birth-weight infants fed medium-chain triglycerides compared to long-chain triglycerides (14). However, other studies have reported no significant differences in gastric emptying based on the fatty acid chain length of triglycerides in form (15). In infants, significantly faster gastric emptying was observed with whey-hydrolysate formulas than with casein-predominant formulas (16). 

 Conversely, gastric emptying rates in preterm infants fed casein-predominant formulas were similar to those fed whey-predominant formulas (17). Pharmacotherapy with prokinetic drugs is often necessary in addition to dietary modifications, though robust evidence is lacking due to the rarity of pediatric cases. Domperidone, a dopamine D2 receptor antagonist, has both antiemetic and prokinetic properties. It enhances the propagation of antral and duodenal contractions, thereby promoting gastric emptying. Erythromycin, a macrolide antibiotic and motilin agonist, improves gastric emptying by increasing antral contractions and enhancing antro-duodenal coordination, and reducing fundic volume (3, 10, 11).

 Intrapyloric botulinum toxin (BTX) injection (IPBI) was administered into the 4 quadrants of the pylorus (25 U per quadrant). Pyloric intervention should be considered when children with gastroparesis continue to experience symptoms that adversely affect their nutritional status or quality of life, despite receiving medical treatment, including dietary modifications and pharmacotherapy (4). 

 Botulinum toxin is a neurotoxin produced by the anaerobic gram-positive, spore-forming bacterium Clostridium botulinum, that elaborates eight antigenically distinguishable exotoxins (A, B, C1, C2, D, E, F, and G) (18, 19). 

 The toxin works by blocking the release of the neurotransmitter acetylcholine, the principal neurotransmitter at the neuromuscular junction. When administered intramuscularly, botulinum toxin acts at the neuromuscular junction, causing muscle paralysis by inhibiting acetylcholine release from presynaptic motor neurons (18). Botulinum toxin type A (BTXA) has been widely studied and recommended for over 25 years in clinical settings for the treatment of lower and upper limb hypertonia in children with cerebral palsy (CP) (19-21). 

 Data on the use of IPBI in pediatric populations with gastroparesis is quite limited (22-24). The beneficial effect of IPBI on gastric emptying in children was first reported by Woodward et al. (22) who described a 9-year-old boy with a 4-5-year history of upper abdominal pain related to eating. Before IPBI, a gastric emptying study revealed a marked delay, with a half-time for the liquid phase of 266.6 minutes. Following the injection, his symptoms dramatically improved, and a repeat gastric emptying study three weeks later showed that the half-time had decreased to 97.6 minutes. A single-center retrospective study of children aged 2 months to 5 years who received IPBI from May 2007 to June 2019 revealed that 47 (82%) children experienced partial improvement and 10 (18%) patients achieved complete resolution of symptoms following their first IPBI. There was a notable enhancement in feeding, with more patients receiving at least some oral feeds and fewer patients relying solely on post-pyloric feeds after IPBI compared to before IPBI (23). 

 Another retrospective study at a single tertiary care center involving 45 children reported that 12 (40%) children were asymptomatic, 15 (50%) had a moderate response and 3 (10%) had a mild improvement after IPBI. The median duration of the effect of the first IPBI was 3.0 months (95% CI 1.2-4.8 months) (24).

## Conclusion

 This case highlights the potential of intra-pyloric botulinum toxin injection (IPBI) as a treatment option in pediatric gastroparesis unresponsive to standard treatments. IPBI demonstrated significant symptomatic relief and improvement in gastric emptying. Its use underscores the need for further research to define its role in clinical practice, evaluate long-term outcomes and establish evidence-based guidelines for managing pediatric gastro-paresis.
